# Fruit as Food

**Published:** 1889-05

**Authors:** 


### FRUIT AS FOOD.

Taken in the morning, fruit is as helpful to digestion as it is refreshing. The newly awakened function finds in it an object of such light labor as will exercise without seriously taxing its energies, and tissues of the stomach acquire at little cost a gain of nourishment which will sustain those energies in latter and more serious operations. It is an excellent plan, with this object in view, to add a little bread to the fruit eaten. While admitting its possession of these valuable qualities, however, and while also agreeing with those who maintain that in summer, when the body is, at all events, in many cases, less actively employed than usual, meat may be less, and fruit or vegetables more freely used as a food ; we are not prepared to allow that even then exclusively vegetarian regimen is that most generally advisable. Meat provides us with a means of obtaining albuminoid material, which is indispensable, in its most easy assimilable form. It affords us in this material not only an important constituent of tissue growth, but a potent excitant of the whole process of nutrition. It has, therefore, a real definite, and great value in the ordinary diet of a man, and the wholesomeness of fruit combined with farinaceous food as an alternative dietary is not so much an argument in favor of the vegetarian principal, as a proof that seasonable changes in food supply are helpful to the digestive processes and to nutritive changes in the tissues generally.—*London Lancet.*

